# Correction: *Systemic inflammation and suicide risk: cohort study of 419 527 Korean men and women*

**DOI:** 10.1136/jech-2017-210086corr1

**Published:** 2018-11-08

Batty GD, Jung KJ, Lee S, *et al*. Systemic inflammation and suicide risk: cohort study of 419 527 Korean men and women. *J Epidemiol Community Health* 2018;72:572–4. doi: 10.1136/jech-2017-210086.

The open access licence type has been updated to CC BY 4.0 since this article first published.

